# The Renaissance of Unicompartmental Knee Arthroplasty appears rational – A radiograph-based comparative Study on adverse Events and patient-reported Outcomes in 353 TKAs and 98 UKAs

**DOI:** 10.1371/journal.pone.0257233

**Published:** 2021-09-16

**Authors:** Magnus Tveit

**Affiliations:** Department of Orthopedics, Skåne University Hospital, Clinical Sciences, Lund University, Lund, Sweden; University Hospital Leipzig, GERMANY

## Abstract

**Purpose:**

Total knee arthroplasty (TKA) and unicompartmental knee arthroplasty (UKA) are both considered suitable for antero-medial osteoarthritis and spontaneous osteonecrosis of the knee. National registry data are consistent in showing higher revision rates for UKA. Adequately adjusted, these findings may be challenged by differences in adverse events and patient-reported outcomes, as both can have serious long-term implications. Based on preoperative radiographs, the aim was to retrospectively compare the two principle surgeries in these respects.

**Methods:**

All TKA procedures in 2016 in one Swedish county council were, according to certain radiograph-based consensus criteria, visually evaluated for medial UKA suitability. Then, using different regression models, they were compared with the corresponding medial UKAs performed in 2015–2017 regarding complications and patient-reported outcomes one year after surgery.

**Results:**

The UKA group showed an 82% reduced risk (OR 0.2; 95% CI 0.0–0.6) of any complications, whereas the 55% reduced risk of severe complication did not reach statistical significance (OR 0.5; 95% CI 0.1–2.1). These findings corresponded in high-volume surgeries to an absolute complication rate of 0% in the UKA group and 10% in the TKA group (p = 0.005) and to a severe complication rate of 0% and 5% respectively (p = 0.05). Though no differences were seen in any general patient-reported outcomes, the pain and function based OMERACT-OARSI responder criteria indicated in both around a 60% better chance of any response (OR 1.6 CI % 0.6–4.5) and a high response (OR 1.6; 95% CI 0.7–3.4) in the UKA group.

**Conclusion:**

No differences were shown in patient-reported outcomes but a clear difference in risk of complications, favoring the UKA procedure.

## Introduction

For many years unicompartmental knee arthroplasty (UKA) was the standard treatment of choice for any knee osteoarthritis, as was total knee arthroplasty (TKA) after that [1]. The former has again gained popularity [1–4]. The causes may include potential consequences facing a TKA revision [1] and that patient satisfaction after a TKA has not yet reached that of a THA [2, 4]. Antero-medial osteoarthritis (AMOA) and spontaneous osteonecrosis of the knee (SONK) are both considered suitable for either TKA or UKA. With that said, if any, to whom and why consider a UKA still seems very much up for debate [5].

Indisputably, national registry data are consistent in showing higher revision rates for UKA [1–4]. However, the author argues that a TKA-UKA comparison is more complex and nuanced. For example, (surgeon) threshold for revision solely caused by patient dissatisfaction within one year is reported five times higher for UKA compared with TKA [6], as is the risk of revision for any reason if the surgeon does not reach enough volume [7] and/or usage [8]. Furthermore, revision is only one of many severe complications, also referred to as adverse events, associated with any arthroplasty. One study, matching 25,334 UKA with 75,996 TKA, found a 0.5 times risk in prosthetic joint infection, and even lower risk in myocardial infarction, venous thromboembolic events, and cerebrovascular events for the UKA procedure within one year of surgery [9]. A 0.5 times risk in prosthetic joint infection for the UKA procedure was recently repeated in a propensity-score-matched study of 10,494 cases [10].

UKA has shown better range of motion (ROM) [11–13] and more resemblance to the native knee in patients who have both articulations [12, 13]. Outcome regarding pain and function using traditional patient-reported outcome measures (PROMs) seem less conclusive, as case-control studies repeatedly have shown no between-group differences [14–16]. The same applies to four out of five as-yet published randomized control trials (RCTs) [11, 17–19], only Newman et al. have shown a long-term advantage of UKA over TKA [20].

The likelihood of registry-based studies being non-adjusted [7, 8], with a clear focus on first-time revision, and cohort studies being more diverse and biased may contribute to the lack of consensus, which seems unacceptable considering that around 3 million knee arthroplasties are performed globally each year. Therefore, the primary objective of this retrospective radiograph-based study was to determine any adjusted between-group differences in TKA and UKA regarding all potential adverse events within one year of surgery. The secondary objective was to evaluate patient-reported outcomes in the same manner. The author hypothesized the UKA procedure to be superior in both aspects.

## Materials and methods

**Fig 1:**
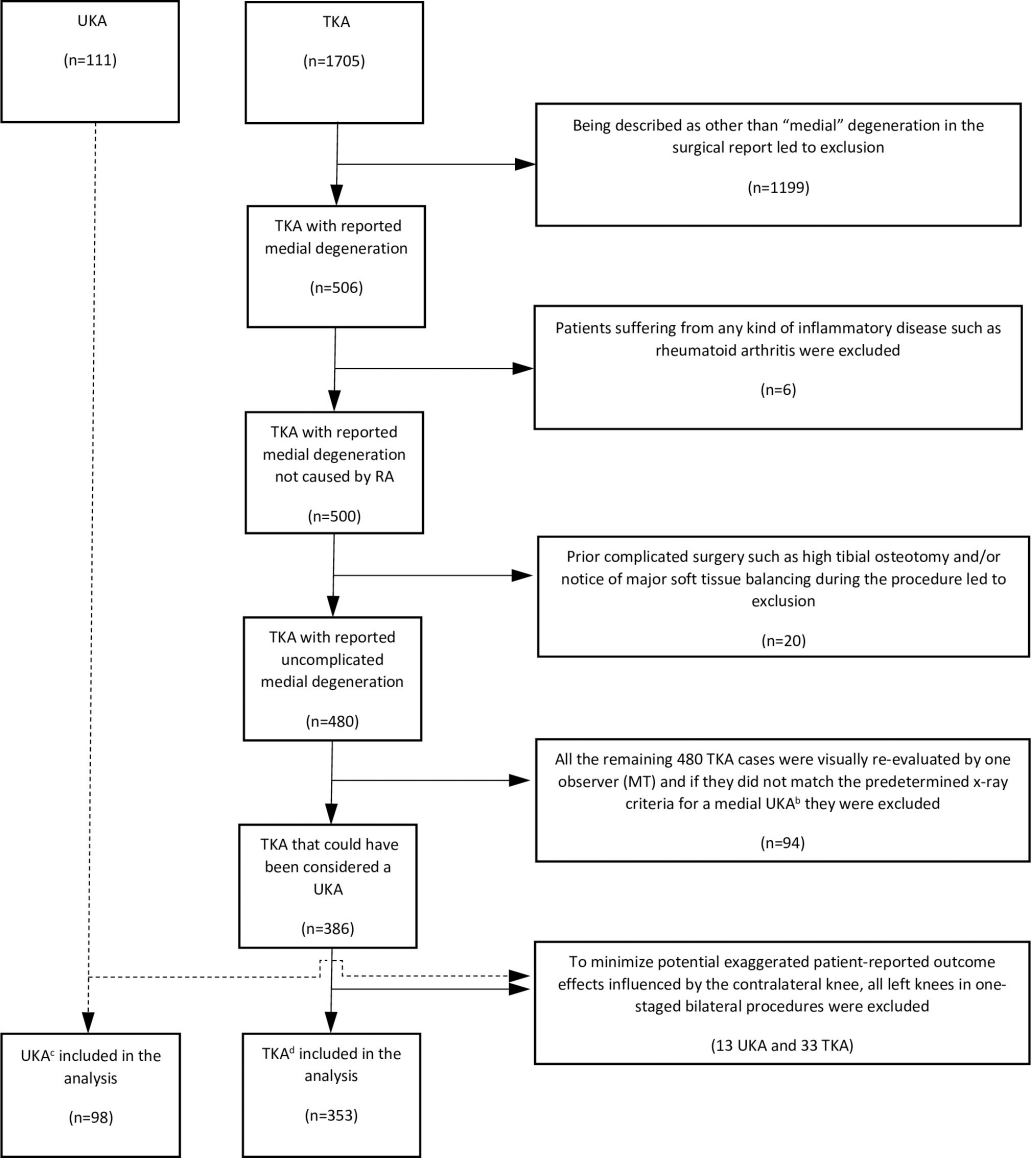
Flowchart of the 451 patients included in the study (and reasons for exclusion)^a^. ^a^ All the primary UKAs performed between 2015 and 2017 and all the primary TKAs performed in 2016 within *Region Skåne*, the southernmost county council of Sweden, were screened for eligibility. To the author´s knowledge, neither any gliding in indication nor altering of the surgical technique occurred during this timeframe. ^b^ In accordance with the 2D radiograph-based consensus criteria for medial UKA [21], the degeneration on the medial side should present bone-on-bone, have indirect signs of functionally intact ACL, and show full thickness cartilage in the lateral compartment, otherwise be excluded. (Additionally, in the absence of medial/lateral stress x-rays, more than 15 degrees of varus deformity, translation in the coronal plane, and/or lateralization of the patella in the skyline projection also led to exclusion from further analyses in this study.). ^c^ 70 LINK® Sled prosthesis; 28 Triathlon® PKR, Stryker. ^d^ 318 Triathlon®, Stryker; 34 P.F.C.® Sigma®, DePuy Synthes; 1 Vanguard® XP, Zimmer-Biomet.

This study was radiograph-based and designed as a case-control. The first step was to scan all the UKA surgical reports between 2015 and 2017 together with all the equivalent TKAs in 2016 within Region Skåne, the southernmost county council of Sweden, for the diagnoses AMOA and SONK. This was made possible as all the regional hospitals share the same searchable medical software platform as well as radiology archives (Fig 1). The different time spans of the procedures were due to the low number of UKA procedures performed before the most recent rise in popularity. (To the author’s knowledge, neither any gliding in indications nor any difference in surgical technique occurred during this period.) It was also the most recent timeframe possible for a one-year follow-up based on the experience that it takes about one year for each hospital to confirm its data.

The second step was to visually evaluate all the preoperative radiographs of each TKA procedure according to certain radiographic consensus criteria [21] to determine suitability for a UKA (Fig 1), as the AMOA/SONK diagnoses alone were not considered adequate proof. As demonstrated in Table 1, although between-group differences were found in several patient characteristic variables, the largest difference was observed in surgeon-volume. All patient characteristics that differed significantly between groups were adjusted for in a stepwise multivariate analysis. A separate univariate analysis was conducted exclusively in high-volume procedures.

**Table 1:** Between-group analyses of patient characteristics and perioperative settings in the TKA and UKA subgroups^a^.

	TKA (*n* = 353)	UKA (*n* = 98)	Between-group difference	
	mean ± SD or %	mean ± SD or %	mean (95% CI)	*P* value
Age (years)	69.8 ± 8.8	67.7 ± 8.5	-2.1 (-4.6 to -0.1)	**0.03**
Female (%)	55.5	42.9	-12.7 (-1.4 to -23.9)	**0.03**
Height (cm)	170.3 ± 9.9	172.7 ± 10.7	2.4 (-0.0 to 4.8)	0.05
Weight (kg)	84.8 ± 15.6	83.0 ± 15.7	-1.8 (-5.3 to 1.7)	0.32
Body mass index (kg/m^2^)	29.2 ± 4.9	27.7 ± 4.0	-1.5 (-2.4 to -0.5)	**0.003**
ASA class ≥3 (%)	16.4	8.2	-8.3 (-1.5 to -15.0)	**0.03**
Charnley class C (%)	45.4	31.9	-13.5 (-2.5 to -24.5)	**0.03**
Osteoarthritis, medial^b^ (%)	97.5	95.9	-1.5 (-5.8 to 2.8)	0.44
Osteonecrosis, medial (%)	2.5	4.1	1.5 (-2.8 to 5.8)	0.44
Other degenerative knee diseases (%)	0.0	0.0	-	-
One-stage bilateral procedure (%)	8.8	11.2	2.4 (-4.6 to 9.4)	0.47
PS procedure (TKA) (%)	0.0	-	-	-
Lateral procedure (UKA) (%)	-	0.0	-	-
High-volume^c^ surgery procedure (%)	94.3	43.9	-50.5 (-40.2 to -60.7)	**<0.001**

^a^ Descriptive data are presented as unadjusted means with standard deviations (SD) or as proportions (%). The between-group differences are presented as means with 95% confidence intervals (95% CI) and p values using Welch’s test and Likelihood Ratio Test (or when violated Fisher’s exact test) respectively.

^b^ Although radiologically impossible to separate from one another, they were described either as “primary” (99.7% TKA / 96.8% UKA) or “secondary” (0.3% TKA / 3.2% UKA) osteoarthritis in the surgical report.

^c^ In this study defined as ten or more of the specific procedure performed each year. In total 376 procedures consisting of 333 TKAs (301 Triathlon®, Stryker; 31 P.F.C.® Sigma®, DePuy Synthes; 1 Vanguard® XP, Zimmer-Biomet) and 43 UKAs (LINK® Sled prosthesis) respectively.

At one-year follow-up, the patient-reported outcome evaluations were divided into three separate areas based on the questionnaires used in Region Skåne at the time; general health (*EQ-5D-3L* [22] *index* and *EQ-VAS*), combined general and knee health (*KOOS* [23]), and met expectations regarding the overall treatment (*Satisfaction VAS*). Intra- and inter-group comparisons of change in PROMs from before to one year after surgery and whether minimum clinically important difference (MCID) [23], also referred to as minimal important change (MIC), had been reached, were also assessed.

Separately, knee specific pain and function dimensions were evaluated (*OMERACT-OARSI responder criteria* [24], here derived from KOOS as it includes WOMAC) as were complications/adverse events (equaled having a plausible causal association with the procedure, defined by the Swedish Knee Arthroplasty Registry [1]). Information of any re-operation, including revision, was confirmed by reading each surgical report.

### Statistics

Descriptive data are presented as unadjusted means with standard deviations (SD) or as proportions (%) and between-group differences as means with 95% confidence intervals in parentheses (95% CI) and p values. The univariate analyses were conducted using Welch’s test and Likelihood Ratio Test (or if violated Fisher’s exact test) respectively. The paired sample t-test was used for the intra-group MCID analysis. The adjusted data are presented as regression coefficients (β) and odds ratios (OR) respectively, with 95% confidence intervals in a manner similar to that above. The stepwise multivariate analyses were conducted using multiple linear or logistic regression, depending on whether the data was numerical or categorical. To enable comparisons of summarized scores and/or dimensions from different scores, with different scales and skewness, numerical variables were also converted into standardized values (z-scores).

A p value less than 0.05 was considered statistically significant. Furthermore, the statistical considerations are based on unequal sample sizes. Although both groups were considered large enough to run parametric tests, each group’s variables were still confirmed visually regarding normality by Q–Q probability plots. Data analyses were conducted using SPSS^®^ Statistics, Version 25 (IBM^®^, Armonk, New York, USA).

### Ethical approval

This observational study (case-control designed) was carried out in compliance with the 7^th^ version (2013) of the Helsinki Declaration and according to the STROBE Statement (www.strobe-statement.org). The study was approved by the Swedish Ethical Review Authority (2020–02001) as of June 17, 2020 and by the regional Ethical Review Board of Region Skåne (173–20) as of Sep 29, 2020.

## Results

The UKA group showed a significant 82% lower risk (OR 0.2; 95% CI 0.0–0.6) of any complications, whereas the observed 55% lower risk of severe complications did not reach statistical significance (OR 0.5; 95% CI 0.1–2.1) (Table 2B and Table 3). (For any complication, only type of implant (p = 0.007) and surgeon-volume (p = 0.001) had confounding effects in the regression model.) The pain and function algorithms of the OMERACT-OARSI responder criteria showed a trend towards the UKA group having around a 60% higher chance of reaching both any responder level (OR 1.6; 95% CI 0.6–4.5) as well as a high responder level (OR 1.6; 95% CI 0.7–3.4) compared with the TKA group (Table 2B).

**Table 2:** Unadjusted absolute and adjusted relative between-group comparisons of patient-reported outcomes and complications in the TKA and UKA subgroups^a^.

	TKA / UKA	Unadjusted			Adjusted beta coefficient and odds ratio in different regression models (TKA as ref.)		
		TKA	UKA		Age	Age, ASA class, BMI, Charnley class, and gender	Age, ASA class, BMI, Charnley class, gender, and high-volume surgery
**A. General and combined PROMs**	N/N	mean ± SD	mean ± SD	P	β (95% CI)	β (95% CI)	β (95% CI)
**At 1-year follow-up**							
Overall satisfaction (VAS)^b^	311/72	18.27 ± 21.23	21.07 ± 23.19	0.35	2.71 (-2.87 to 8.29)	4.41 (-1.27 to 10.09)	3.51 (-2.96 to 9.98)
EQ-5D *index*	308/73	0.783 ± 0.218	0.798 ± 0.194	0.57	0.02 (-0.04 to 0.07)	-0.01 (-0.06 to 0.04)	-0.03 (-0.09 to 0.03)
EQ-VAS	312/74	76.46 ±19.26	78.89 ± 18.98	0.33	2.61 (-2.29 to 7.51)	0.22 (-4.45 to 4.89)	0.66 (-4.63 to 5.95)
KOOS *Symptoms*	309/74	77.90 ± 16.38	80.79 ± 14.87	0.14	3.59 (-0.43 to 7.61)	2.37 (-1.75 to 6.49)	2.48 (-2.20 to 7.17)
KOOS *Pain*	309/74	80.64 ± 18.42	82.72 ± 15.44	0.32	2.27 (-2.29 to 6.84)	0.51 (-4.10 to 5.13)	1.70 (-3.55 to 6.95)
KOOS *ADL*	309/74	80.17 ± 17.64	81.31 ± 19.07	0.64	1.13 (-3.45 to 5.71)	-0.58 (-4.96 to 3.79)	-0.15 (-5.13 to 4.84)
KOOS *Sport Rec*	309/74	41.89 ± 26.48	45.07 ± 24.66	0.33	3.84 (-2.79 to 10.46)	0.23 (-6.41 to 6.88)	-0.78 (-8.34 to 6.77)
KOOS *QOL*	309/74	65.67 ± 22.90	65.95 ± 21.40	0.92	1.19 (-4.48 to 6.85)	-0.70 (-6.45 to 5.04)	-1.43 (-7.97 to 5.11)
**Difference (from before surgery to one year after surgery)**							
EQ-5D *index*	287/71	0.325 ± 0.349	0.261 ± 0.304	0.13	-0.07 (-0.16 to 0.02)	-0.05 (-0.14 to 0.04)	-0.07 (-0.18 to 0.03)
EQ-VAS	289/71	8.64 ± 24.36	7.55 ± 23.70	0.73	-1.69 (-7.98 to 4.60)	-1.43 (-7.83 to 4.96)	-1.35 (-8.62 to 5.92)
KOOS *Symptoms*	285/72	28.35 ± 20.88	29.23 ± 21.52	0.76	0.46 (-4.99 to 5.90)	1.99 (-3.43 to 7.40)	2.44 (-3.72 to 8.60)
KOOS *Pain*	285/72	40.50 ± 21.10	41.52 ± 20.63	0.71	0.41 (-5.00 to 5.82)	1.01 (-4.47 to 6.50)	3.12 (-3.10 to 9.35)
KOOS *ADL*	285/72	34.56 ± 20.19	33.96 ± 21.52	0.83	-1.05 (-6.35 to 4.25)	-0.57 (-5.93 to 4.79)	0.63 (-5.47 to 6.72)
KOOS *Sport Rec*	285/72	29.74 ± 25.92	29.03 ± 24.60	0.83	-0.37 (-7.04 to 6.30)	-1.52 (-8.28 to 5.23)	-2.45 (-10.14 to 5.23)
KOOS *QOL*	285/72	43.69 ± 23.50	41.68 ± 22.62	0.50	-1.71 (-7.77 to 4.35)	-1.03 (-7.19 to 5.13)	-1.26 (-8.27 to 5.75)
**B. Knee specific PROMs and complications**	N/N	%	%	P	OR (95% CI)	OR (95% CI)	OR (95% CI)
OMERACT-OARSI *Responder*^*c*^	283/72	88.0	90.2	0.59	1.21 (0.51 to 2.88)	1.38 (0.57 to 3.36)	1.60 (0.57 to 4.46)
OMERACT-OARSI *High responder*^*c*^	283/72	77.7	77.8	0.99	0.96 (0.51 to 1.79)	1.07 (0.56 to 2.04)	1.56 (0.71 to 3.43)
Complication^d^	353/98	10.5	5.1	0.08	0.46 (0.18 to 1.22)	0.54 (0.20 to 1.48)	**0.18 (0.05 to 0.62)**
Severe complication^d^	353/98	4.8	4.0	0.76	0.89 (0.29 to 2.73)	0.91 (0.28 to 2.98)	0.45 (0.10 to 2.10)

^a^ Data are presented as number of individuals (N). Unadjusted absolute values are presented as means with standard deviations (SD) or as proportions (%). The analyses were done by either of Welch’s test and Likelihood Ratio Test (or when violated Fisher’s exact test). The adjusted relative values are presented as beta coefficients (β) or odds ratios (OR) with 95% confidence intervals (95% CI) in parentheses, depending on if numerical or categorical variables. The regression analyses in different models were done by multiple linear regression or multiple logistic regression, depending on variable category.

^b^ Met expectation of the overall treatment (VAS 0–100, best–worst).

^c^ The OMERACT-OARSI pain and function criteria are based on WOMAC (which in turn can be derived from KOOS): A “high responder” is defined as an individual with ≥50% total improvement and ≥20% improvement in the pain and function dimensions, whereas a “non-responder” is defined as an individual with <20% total improvement or <10% improvement in the pain and function dimensions. A “responder” scores in-between these.

^d^ Defined as having a plausible causal association with the procedure. Examples of a severe a complication, also referred to as an adverse event (AE), included venous thromboembolism, myocardial infarction, cerebrovascular accident, infection, revision, and early mortality. (A detailed definition of the AE variables used in this study is described in the Swedish Knee Arthroplasty Register’s 45^th^ Annual Report [1].)

**Table 3:** Proportions of severe complications^a^ found in each subgroup within one year after surgery.

	TKA (*n* = 353)		UKA (*n* = 98)	
	n	%	n	%
**Perioperative events**	**2**	**11.8**	**0**	**0.0**
Fracture	2	11.8	-	-
**Reoperation**	**5**	**29.4**	**2**	**50.0**
Contraction (→mobilization)	1	5.9	1	25.0
Pain (→revision)	1	5.9	1	25.0
Deep infection (→revision)	3	17.6	-	-
**Cardiovascular**	**6**	**35.3**	**2**	**50.0**
Gastrointestinal bleeding	1	5.9	-	-
Pulmonary thromboembolism	2	11.8	-	-
Myocardial infarction	2	11.8	2	50.0
Cerebrovascular accident	1	5.9	-	-
**Death**	**4**	**23.5**	**0**	**0.0**
**Total**	**17**	**100.0**	**4**	**100.0**

^a^ Corresponded to adverse events as defined in the Swedish Knee Arthroplasty Register’s 45^th^ Annual Report [1].

No between-group differences were detected in any of the general health related patient-reported outcome variables, neither unadjusted nor when adjusted for potential confounders in different regression models (Table 2A). However, all patients were significantly improved from before surgery to one year after surgery in each group, including MCID for all five KOOS dimensions (TKA 21.1–43.4 points, all with p values <0.0001; UKA 26.3–43.6 points, all with p values <0.001).

When looking exclusively at high-volume surgeries, there was a statistically significant absolute difference in any complication between groups (0% in the UKA group and 10% in the TKA group, p = 0.005), whereas the corresponding absolute severe complication rates equaled 0% and 5% respectively (p = 0.05). Most complications that had not reached a “severe” level consisted either of superficial surgical site infections or deep vein thrombosis. The 5% severe complications seen within one year after TKA surgery comprised 4 deaths, 4 revisions, 2 myocardial infarctions, 2 cerebrovascular lesions, 2 perioperative fractures, 1 gastrointestinal bleeding and 1 reoperation caused by knee contraction.

No absolute between-group differences were seen in any of the used PROMs, at 1-year follow-up (EQ-5D index p = 0.99; EQ-VAS p = 0.58; KOOS p = 0.57–0.99; Satisfaction VAS p = 0.56) nor from before surgery to one year after surgery (EQ-5D index p = 0.52; EQ-VAS p = 0.71; KOOS p = 0.41–0.97). No overall trend favoring either group was seen, in fact, after the numerical scales were converted into standardized scores and the overall patient-reported outcomes of three equally weighted structures had been summarized–(i) general health (EQ-5D index and EQ-VAS), (ii) combined general and knee health (KOOS), and (iii) degree of met overall expectations (Satisfaction VAS)–the mean UKA z-score turned out 0.00 (ns). Even here the pain and function weighted OMERACT-OARSI responder criteria showed a trend towards an absolute better knee-related outcome in the UKA group with 93% “responders” vs 88% in the TKA group (ns) and for “high responders” 83% and 79% respectively (ns).

## Discussion

To the author’s knowledge, this is the largest radiograph-based study ever undertaken to compare TKA with UKA, adjusted for confounders, regarding all adverse events and patient-reported outcomes within one year of surgery. The most important finding of the present study was a significant between-group difference in complications with an 82% lower overall risk in the UKA group.

The current study focused on all potential adverse events, as they are often overshadowed by first-time revision data in the national registries. A 1-year follow-up was considered sufficient as by definition adverse events such as pulmonary thromboembolism, myocardial infarction, cerebrovascular accident, and death should have a close and plausible association to the surgery. Indisputably, the adverse events found in the current study all imply risk of long-term consequences.

### Adverse events

Yet, uniform register data show higher revision rates for UKA than TKA [1–4]. Furthermore, two [1] to four [4] times higher revision rates (to TKA) were reported if UKA was index surgery compared with primary TKA. The same is true of corresponding inferior patient-reported outcomes [25]. With that said, superior patient-reported outcomes have been shown in UKA-TKA compared with TKA-TKA revision [16], probably caused by a lower risk of needing augments, stems, or more constrained revision implants in the former scenario [1, 25]. Therefore, UKA as an index operation may well be favored over TKA if “revision implant”, or even worse, were to be endpoint variables [1]. This may especially concern SONK as registry data from Australia has shown 26% higher 10-year revision rates for osteonecrosis compared with osteoarthritis in TKA [3]. As was also demonstrated in this study, revision only counts for one of many adverse events that would be defined as severe complications [1, 9, 10]. Although complications should foremost be valued on a personal level, they are indisputably also very much linked to the overall costs. UKA has been reported more cost-effective than TKA in registry-based studies [26–29]. Mid-term results (five years) from the largest RCT so far are in line with these results [17]. The initial cost savings seem to persist for life [29], where surgeon-usage appears to have significant impact on the overall cost-effectiveness of the UKA procedure [27]. Correspondingly in this study, it was not until surgeon-volume was included in the regression model that the relative risk of complications significantly decreased in the UKA group (Table 2B).

### PROMs

Most studies have not been able to show any between-group differences in PROMs when comparing TKA and UKA [11, 14–19]. One explanation could be, somewhat surprisingly, that out of different osteoarthritic disease patterns AMOA is indicated to achieve the best improvements when exclusively analyzing TKA procedures [30]. Other plausible explanations to the many non-significant findings in the literature could be that traditional PROMs are either affected by too many confounding factors in their general health-weighted output designs or related to their moderate ability to differentiate well-functioning patients. To a certain extent, both effects may have been present in this study as it also used rather general health-weighted scores.

A recent matched study of 135 TKAs and 135 UKAs reported both significantly improved pain and function, activity level, and satisfaction scores at 1-year follow-up for the UKA using the *new* Knee Society Score (KSS), the University of California Los Angeles (UCLA) activity-level score, and a Likert satisfaction scale respectively [31]. The Total Knee Questionnaire (TKQ) and the Forgotten Joint Score (FJS-12) have both demonstrated low ceiling effects in patient satisfaction measures and found UKA to be superior in this aspect [32, 33]. In a systematic review comparing TKA and UKA regarding knee awareness (FJS-12) five studies (930 patients) were eligible for a Forest plot meta-analysis two years after surgery and reported a mean difference of 7.6 (95% CI 3.7–11.6), favoring the UKA procedure [34].

### Volume

Arguably, UKA constitutes one of two main surgical options for AMOA and SONK, but what percentage should be recommended? In the literature a span of around five [35] to fifty [36] percent is reported. Recent findings have reported significantly lower revision rates if both a yearly surgeon-volume of more than 12 cases [7] and surgeon-usage of more than 20% [8] are reached, then even suggested to reach similar revision rates as for TKA [8], arguments not contradicted in this study (Table 2B). (During the data collection for this article, according to the stipulated radiological consensus criteria [21], a minimum of 27.4% of all knee arthroplasties performed within Region Skåne in 2016 were determined to be suitable for a UKA (as only the TKA procedures reported “medial” were evaluated).) Newer findings on age, chondrocalcinosis, weight and patellofemoral disease [21, 36] have suggested a broadening of the original indications [35]. Accordingly, around 10% of the UKA cases in high-volume surgeons appear to be on the lateral side with similar performance as on the medial side [37]. Furthermore, a meta-analysis comprising five cohort studies found no difference in functional outcome or revision rates when comparing UKA with and without patellofemoral joint arthritis [38]. Beard et al. found no relationship between anterior knee pain (AKP) and degeneration, nor between preoperative AKP and postoperative function score in UKA [39]. There is, however, consensus that traumatic ACL injuries, high tibia osteotomy (HTO) and inflammatory arthritis should be considered non-recommendable for UKA surgery [21].

### Age

Although two studies have reported younger patients’ performance as inferior after TKA than after UKA [40, 41], these findings should not be translated into the somewhat persistent view that the younger population is more suitable for a UKA than the elderly population. According to the literature this is far from correct. In an effort to save bone stock, the younger population may even be better off with a HTO [42], whereas two recent meta-analyses have shown UKA to be especially favorable in the elderly population [43, 44]. As was also shown in this study, irrespective of age (Table 2B), there is solid evidence that UKA is associated with a substantially lower risk of adverse events than TKA [9]. Patients older than 75 years were reported to have lower rates of postoperative transfusions, greater postoperative range of motion, and higher levels of activity at the time of discharge if operated on with a UKA compared with a TKA [15], which speaks further in favor of the UKA procedure in the elderly population. Another study showed that patients older than 75 years had no between-group difference in revision rate at five years [15]. Data from the Swedish Knee Arthroplasty Registry, in analogy found a two-times better 10-year UKA survival in those above 75 years of age compared to those below 65 years of age [1]. UKA is also reported to be more cost-effective than TKA as the initial health improvements appear to be maintained for the elderly patients, something that is not as evident for the younger patients depending on the lifetime risk of revision [29]. A study of the Norwegian Joint Registry concluded UKA to be a more cost-effective strategy than TKA for the elderly low-demand population as long as the annual probability of revision was below four percent [28].

### Strengths

To the author’s knowledge, this is the largest radiograph-based comparative study ever undertaken that addresses adverse events and patient-reported outcomes between TKA and UKA, while also adjusting for patient characteristics and perioperative settings. The study is authentic in that it comprises several surgeons at five centers, yet with a minimum of confounding factors as it was conducted during a short period of time within one county council in the south of Sweden, i.e., demographics, patient characteristics, and perioperative routines were expected to be very similar. Nevertheless, the fact that it was adjusted for known confounding patient characteristics such as age, ASA class, BMI, Charnley class, and gender makes the data robust (Tables 1 and 2). An important strength of this study is the fact that only one single individual evaluated all the radiographs, rolling out any kind of inter-observer bias. The response rates were considered high, especially among the high-volume cases (TKA 82–100% and UKA 90–100%, depending on variable). Finally, the study is unique in that it provides evidence of the importance of adjusting for surgeon-volume (which could shed some light on the so often diametrical differences in results when comparing cohort with registry-based studies).

### Weaknesses

First, the study was not randomized, but given the retrospective approach, it was possible to conduct a large multi-center comparative study with similar perioperative routines, demographics, and adjustments for potential confounders. One other limitation would be the strict radiograph-based inclusion/exclusion criteria [21] used, without evaluating any medical notes regarding the choice of implant prior to surgery based on center and/or surgeon preferences. Furthermore, although the consensus criteria include both evaluation of the cartilage and indirectly the viability of the ACL [21], the suitability for a UKA should finally be confirmed during surgery, which was obviously not the case in this study. With that said, conventional lateral x-ray alone is proved 93% sensitive and 96% specific in determining UKA suitability for medial degeneration [45], and has also shown to be more reliable than both MRI and clinical examination for assessing ACL viability in OA [46]. Finally, the fact that the approximately 60% higher chance of achieving a better pain and function response, according to OMERACT-OARSI responder criteria, did not reach statistical significance in the UKA group indicates a type II error.

### A personal reflection

For doctor and patient alike a risk/reward discussion is often meaningful but requires data to be presented in a relevant manner, of which the set of OMERACT-OARSI responder criteria is one such attempt. A clear aim of the presentation in this study was to summarize the results in order to make them useful in such a doctor/patient scenario, both in relative and absolute terms. The author argues that caution should be taken not to exaggerate non-significant summarized overall PROM results, as most traditional scores are either weighted towards or incorporate evaluation of general health, with individual dimensions often overlapping, and thereby risk blurring a specific area of interest. (This fact was deliberately demonstrated in this study by a summarized mean UKA z-score of 0.00 (ns) in the high-volume procedures using the included PROM structures (while both respecting the differences in scale and skewness but assumed equal weight) and at the same time, somewhat contradictory, a trend of both an absolute higher rate of any knee specific “response” (5%) and “high response” (4%), as well as both a significant absolute lower rate of any complications (10%) and severe complications (5%) compared with TKA.)

## Conclusions

This study could not show any differences in patient-reported outcomes but a clear difference in risk of complications favoring the UKA procedure.

Further TKA-UKA evaluations on patient-reported outcome may include other measures such as the FJS-12 which has proven to have a comparable low ceiling effect, and thereby is superior in separating patients with good to excellent outcome. To include type of revision implant rather than solely focusing on revision may also be of value to better understand the overall picture when considering either TKA or UKA as index operation. In the meantime, when considering a UKA procedure, it seems advisable to ensure high enough volume/usage, and not to rule out the elderly.
